# *FERMT2* rs17125944 polymorphism with Alzheimer's disease risk: a replication and meta-analysis

**DOI:** 10.18632/oncotarget.9679

**Published:** 2016-05-27

**Authors:** Qiu-Yue Zhang, Hui-Fu Wang, Zhan-Jie Zheng, Ling-Li Kong, Meng-Shan Tan, Chen-Chen Tan, Wei Zhang, Zi-Xuan Wang, Lin Tan, Jin-Tai Yu, Lan Tan

**Affiliations:** ^1^ Department of Neurology, Qingdao Municipal Hospital, School of Medicine, Qingdao University, Qingdao, China; ^2^ Department of Geriatrics, Qingdao Mental Health Center, Qingdao, China; ^3^ Department of Emergency, Qingdao Municipal Hospital, School of Medicine, Qingdao University, Qingdao, China; ^4^ College of Medicine and Pharmaceutics, Ocean University of China, Qingdao, China

**Keywords:** Alzheimer's disease, FERMT2, polymorphism, association study, meta-analysis, Gerotarget

## Abstract

A recent meta-analysis of genome-wide association studies (GWAS) in population of Caucasian identified a single nucleotide polymorphism (SNP) rs17125944 in the *FERMT2* gene as a new susceptibility locus for late-onset Alzheimer's disease (LOAD). In order to validate the association of the rs17125944 polymorphism with LOAD risk in the northern Han Chinese, we recruited a case–control study of 2338 Han Chinese subjects (984 cases and 1354 age- and gender-matched controls). Our results demonstrated that there was no significant association between the rs17125944 polymorphism and LOAD (genotype: *P* = 0.953; allele: *P* = 0.975). Furthermore, no significant differences were observed in alleles and genotypes distribution after stratification by apolipoprotein E (APOE) ε4 and multivariate logistic regression analysis. We also performed a meta-analysis in 81908 individuals. The meta-analysis showed that the C allele is the risk factor for LOAD in Caucasian group (OR = 1.15, 95 % CI = 1.10–1.20) and combined population (OR = 1.13, 95 % CI = 1.08–1.19). While in Chinese population, the C allele is not associated with increased risk of LOAD (OR = 1.07, 95 % CI = 0.89–1.28). In conclusion, our study showed that the rs17125944 polymorphism in FERMT2 gene might not be association with LOAD in northern Han Chinese population.

## INTRODUCTION

Alzheimer's disease (AD), as a progressive neurological disorder, is a major form of dementia in the elderly. Its two pathological hallmarks in brain are extracellular amyloid plaques, which are deposits of amyloid β (Aβ) peptide, and intracellular neurofibrillary tangles (NFTs), which are formed by the microtubule-associated protein tau [1]. Late-onset Alzheimer's disease (LOAD) is the most frequent form of AD with a strong genetic component [2]. Until now, apolipoprotein E (APOE) is the major risk factor for LOAD, however, it does not explain the whole genetic diversity of AD [3]. Genome-wide associated studies (GWAS) have identified several additional genes that contribute to LOAD risk, including *ABCA7*, *CLU*, *CR1*, *CD33*, *CD2AP*, *EPHA1*, *BIN1*, *PICALM*, and *MS4A* [4, 5]. Recently, 11 new susceptibility loci for AD also have been identified in a meta-analysis of GWAS in individuals of European ancestry [6].

*FERMT2* (as known as kindlin-2) is a member of kindlin protein family, which also contains kindlin-1 and kindlin-3 [7]. Kindlin-2, as an integrin-interacting protein, is ubiquitously expressed and mediates integrin activation and integrin-mediated cell-extracellular matrix (ECM) interaction [7, 8]. A study has been showed that kindlin-2 participates in the angiogenesis of mice and zebrafish and mediating cardiac structure and function [9, 10]. *FERMT2* also promotes breast cancer progression through activating genome instability [11]. In recent study of functional screening in Drosophila, Fit1 and Fit2, as orthologs of human *FERMT2*, were found to modify Tau toxicity [12]. This result suggested that *FERMT2* may be related to LOAD risk by influence Tau-mediated pathological process. The single nucleotide polymorphisms (SNP), rs17125944 in the *FERMT2* gene on chromosome 14q22.1 was firstly found to be associated with LOAD in the completion of meta-analysis with 74,046 individuals from Caucasians. As a new susceptibility locus, rs17125944 reached genome-wide significance in the combined discovery and replication analysis [6]. Then three studies have replicated the association of rs17125944 polymorphism with the risk for LOAD. Ruiz et al. validated the association in Spanish [13], and Xiao et al. verified the association in Southern Han Chinese [14]; however, Jiao et al. failed to replicate the association of rs17125944 polymorphism with LOAD in another Chinese population [15]. Since there were different results about the association of rs17125944 with LOAD risk in Han Chinese [16], it was essential to confirm the genetic association in other Chinese groups. Here, we carried out an analysis about the correlation between rs17125944 polymorphism in *FERMT2* gene and LOAD risk in northern Han Chinese to validate the possible genetic association. We also conducted a meta-analysis of present association studies in different ethnic groups to detect the relationship between rs17125944 polymorphism and LOAD risk.

## RESULTS

### Replication study

The demographic and clinical characteristics of the study participants are presented in Table 1. AD patients were well-matched with control subjects in terms of age and gender. As expected, the MMSE scores in case group were significantly lower than in control subjects. And the presence of the APOE gene ε4 allele increased the risk of LOAD in patients when compared with controls (*P* < 0.001, OR = 2.422, 95%CI = 1.970-2.977). Distributions of genotypes in AD patients and control subjects were in the Hardy-Weinberg equilibrium (*P* > 0.05).

**Table 1:** The characteristics of the study population

Characteristic	Patients with AD (*n* = 984)	Control subjects (*n* = 1354)	*P* value	OR (95% CI)
Age at examination, years; mean ± SD	79.81±6.71	75.50 ± 6.49	0.186^*^	
Age at onset, years; mean ± SD	75.15 ± 6.08			
Gender, n (%)				
Male	406 (41.26%)	610 (45.05%)	0.068	
Female	578 (58.74%)	744 (54.95%)		
MMSE score, mean±SD	11.99 ± 6.20	28.49 ± 1.09	<0.001	
APOE ε4 status, n (%)				
APOE ε4 (+)	280 (28.46%)	191(14.11%)	<0.001	2.422 (1.970-2.977)
APOE ε4 (−)	704 (71.54%)	1163 (85.89%)		

Abbreviations: AD, Alzheimer disease; APOE, apolipoprotein E; CI, confidence interval; MMSE, Mini Mental State Examination; OR, odds ratio; SD, standard deviation.

* *P* value was calculated with the age of onset for late-onset AD and age at examination for control subjects. APOE ε4 (+) refers to subject carrying at least one APOE ε4 allele; APOE ε4 (−) refers to subject carrying no APOE ε4 allele. Differences in the characteristics of age and MMSE score between the two groups were assessed using the Student's t test. Differences in gender and APOE ε4 status between AD patients and control subjects were estimated using the Chi-square test.

The allele and genotype distributions of rs17125944 in cases and controls were showed in Table 2. And there were no significant differences between patients of LOAD and control participants (genotype *P* = 0.953; allele *P* = 0.975, OR = 0.998, 95% CI = 0.868-1.146). Then we stratified these data through the presence/absence of the APOE ε4. However, no significance was observed in both subjects with APOEε4 allele (genotype: *P* = 0.398; allele: *P* = 0.377, OR = 1.151, 95% CI = 0.842-1.572) and without the APOE ε4 allele (genotype: *P* = 0.395; allele: *P* = 0.586, OR = 0.957, 95% CI = 0.816-1.122). Furthermore, by multivariate logistic regression, there was also no association between the rs17125944 polymorphism and LOAD (dominant model: *P* = 0.766, OR = 0.974, 95% CI = 0.822-1.156; additive model: *P* = 0.937, OR = 0.994, 95% CI = 0.863-1.146; recessive model: *P* = 0.647, OR = 1.094, 95% CI = 0.746-1.603) after adjusting for age, gender, and the APOE ε4 allele carrier status (Table 3).

**Table 2:** Distribution of the rs17125944 genotypes and alleles in the controls and the AD cases

	n	Genotypes n (%)				Allele n (%)			
		TT	TC	CC	*P*	T	C	*P*	OR (95%CI)
rs17125944									
Control	1354	808(59.68)	480(35.45)	66(4.87)	0.953	2096(77.40)	612(22.60)	0.975	0.998(0.868−1.146)
AD	984	590(59.96)	344(34.96)	50(5.08)		1524(77.44)	444(22.56)		
APOE ε4(+)									
Control	191	117(61.26)	66(34.55)	8(4.19)	0.398	300(78.53)	82(21.47)	0.377	1.151(0.842-1.572)
AD	280	156(55.72)	114(40.71)	10(3.57)		426(76.07)	134(23.93)		
APOE ε4(−)									
Control	1163	691(59.41)	414(35.60)	58(4.99)	0.395	1796(77.21)	530(22.79)	0.586	0.957(0.816−1.122)
AD	704	434(61.65)	230(32.67)	40(5.68)		1098(77.98)	310(22.02)		

Abbreviations: *P* value was examined using the Chi-square test.

**Table 3:** Multivariate logistic regression in AD cases and controls

SNP	Models	*P* value	OR (95%CI)
rs17125944	Dominant	0.766	0.974 (0.822-1.156)
	Additive	0.937	0.994 (0.863-1.146)
	Recessive	0.647	1.094 (0.746-1.603)

Adjusted for age, gender, and the APOE ε4 allele carrier status. SNP: single nucleotide polymorphism.

### Meta-analysis

**Figure 1:**
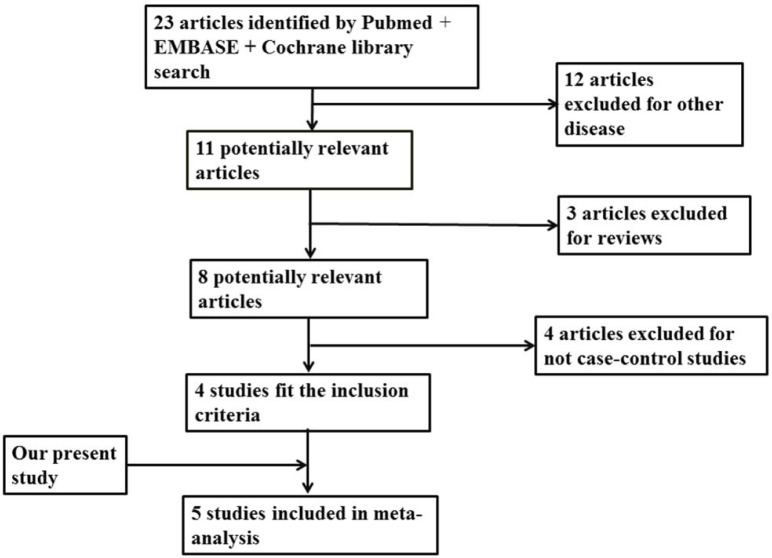
Flow diagram of the study selection

**Figure 2:**
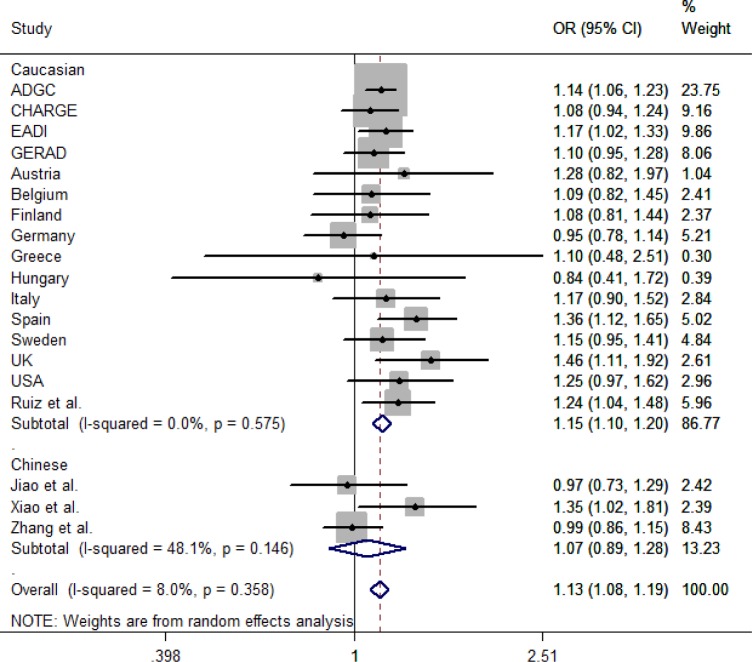
Forest plot of rs17155944 polymorphism associated with late-onset Alzheimer's disease Abbreviations: The squares and horizontal lines correspond to OR and 95 % CI of the definite study, and the area of squares represents statistical weight. The diamond reveals the pooled OR and its 95 % CI. OR, odds risk, CI, confidence interval.

Following the search strategy, we got 4 studies, which met the inclusion criteria (Figure 1). Including our study, all the subjects consisted of 28833 cases and 53075 controls. There are 2 studies in individuals of Caucasian and 3 studies in Chinese population [6, 13–15]. We collected the data from the studies, in which OR and 95%CI were evaluated using additive logistic regression model. By pooling our data and 4 previous studies by subgroup, we conducted a meta-analysis (Figure 2). The results showed that the C allele is the risk factor for LOAD in Caucasian group (OR = 1.15, 95 % CI = 1.10-1.20) and combined population (OR = 1.13, 95 % CI = 1.08-1.19). And the heterogeneity (I^2^ = 8.0%) of these studies is not notable. While in Chinese population, the C allele is not associated with increased risk of LOAD (OR = 1.07, 95 % CI = 0.89-1.28).

## DISCUSSION

*FERMT2*, as known as kindlin-2, participates in integrin activation and cell-cell and cell-matrix adhesion [10]. A study has discovered that the fly ortholog of *FERMT2* can modulate Tau toxicity [12]. It suggests that *FERMT2* might play a role in the pathological process of AD through Tau-mediated neuronal injury. In 2013, a meta-analysis of 74 046 samples reported by the International Genomics of Alzheimer's Disease Project (IGAP) firstly found the susceptibility loci rs17125944 in *FERMT2* gene for LOAD [6]. Different from the effect in the population of Caucasian, our current study showed no association of LOAD risk with the rs17125944 polymorphism in a northern Han Chinese population. Furthermore, the meta-analysis of related studies showed that rs17125944 polymorphism has no association with LOAD in Chinese.

Our study failed to replicate the association in the northern Han Chinese. There might be some reasons for the replication failure. First of all, different genetic backgrounds between Han Chinese and European ancestry may lead to the opposite results. There is significant difference on the minor allele frequency (MAF) of rs17125944 between Chinese and Caucasians. The MAF of rs17125944 is 24% in Chinese, while the MAF of rs17125944 is 8.0% in Caucasians based on the dbSNP database (http://www.ncbi.nlm.nih.gov/projects/SNP). Meanwhile, multiple genes and environmental factors and gene-environment interactions may affect LOAD risk [2].

Secondly, the meta-analysis in Chinese showed that the rs17125944 polymorphism has no significant association with LOAD (OR = 1.07, 95 % CI = 0.89-1.28) but with a moderate heterogeneity (I^2^ = 48%, *P* = 0.146). Xiao et al. discovered that rs17125944 is associated with LOAD risk (OR = 1.35, 95%CI = 1.02-1.81) in Southern Han Chinese; however, Jiao et al. and our study did not replicate the relationship between rs17125944 and LOAD risk in other Chinese cohorts [14, 15]. The difference of genetic backgrounds in Han Chinese may explain the diversity. A previous study showed that the Chinese population was substructured with several clusters [16]. The MAF of rs17125944 was 22% in Xiao et al. report and the MAF of rs17125944 was 23% in our study, both of which were similar to the MAF of rs17125944 in HapMap database of Chinese Han from Beijing (24%). Meanwhile, the subjects of our study were from Shandong Province (Northern China), while the participants in Xiao's study were enrolled from Shanghai (Southern China). Jiao et al. recruited their subjects from Hunan Province. The diversity in regional environment, culture, and the level of education in studies maybe the sources of heterogeneity. In addition, sample sizes in studies may also contribute to the difference. The sample size (984 cases and 1354 controls) in our study is larger than that in Xiao et al (232 cases and 373 controls) and Jiao et al.'s study (229 cases and 318 controls). All the above factors may be the sources of the heterogeneity.

In conclusion, our study suggests that the rs17125944 polymorphism in FERMT2 gene might not play an important role in the pathology of LOAD in a northern Han Chinese population. And the result needs further confirmation by studies in larger sample sizes and different ethnic groups. Meanwhile, it is necessary to clarify the exact relationship between FERMT2 and Tau toxicity in human.

## MATERIALS AND METHODS

### Replication study

#### Subjects

In our study, there were 984 sporadic LOAD patients (mean age at onset: 75.15 ± 6.08 years; male 406 and female 578) and 1354 healthy control subjects (mean age at examination: 75.50 ± 6.49 years; male 610 and female 744), which were matched for age and gender. All the participants were Northern Han Chinese. The patients of LOAD were recruited from the Department of Neurology at Qingdao Municipal Hospital, and several other hospitals in Shandong Province. The probable AD was clinically diagnosed on the basis of the criteria of National Institute of Neurological and Communicative Disorders and Stroke and the Alzheimer's disease and Related Disorders Association (NINCDS-ADRDA) by at least two neurologists [17]. None of the AD patients reported a family history of dementia or other dementias. The control participants were recruited from the Health Examination Center of the Qingdao Municipal Hospital. The healthy controls were proved healthy through medical history, general examinations, laboratory examinations and Mini Mental State Examination (MMSE) (score≥28) [18]. Informed consent of participants was obtained from individuals or their guardians. And the study has been approved by the Institute Ethical Committee.

#### Genotype analysis

DNA was extracted from the peripheral blood of participants using the Wizard genomic DNA purification kit (Cat. #A1125, Promega, USA) [19]. The rs17125944 polymorphism was genotyped using the means of SNPscan^TM^ kit (Cat#: G0104K, Genesky Biotechnologies Inc., Shanghai, China) [20]. And the kit was a patent-pending technology from Genesky Biotechnologies Inc., which was developed on double ligation and multiplex fluorescent polymerase chain reaction (PCR). Data analysis was obtained using GeneMapper 4.1 (Applied Biosystems, USA). APOE genotype was measured by the improved multiplex ligase detection reaction (iMLDR) method, which was supported from the Shanghai Genesky Biotechnology Company. And the polymerase chain reaction (PCR) primer oligonucleotide sequences were: rs429358/rs7412, forward: CACGGCTGTCCAAGGAGCTG, reverse: GCTGCCCATCTCCTCCATCC.

#### Statistical analysis

The Hardy-Weinberg equilibrium (HWE) was calculated for SNP using genotype data from AD patients and controls. Differences of the characteristics between cases and controls were examined using the Student *t*-test or the Chi-square test. Genotype and allele distributions were compared using the Chi-square test. Differences in allele and genotype distribution between cases and controls were analyzed using logistic regression adjusted for non-genetic covariates and APOEε4 status under various genetic models that were defined as 1(TC + CC) *versus* 0(TT) for dominant, 1(CC) *versus* 0(TT + TC) for recessive, and 0(TT) *versus* 1(TC) *versus* 2(CC) for additive. The p value, odds ratios (OR) and 95% confidence intervals (CI) were accounted. The statistical analyses were performed by SPSS 16.0 software. The criterion for significant difference was *P* < 0.05.

### Meta-analysis

#### Search strategy

In order to find all the articles about investigating the association between *FERMT2* and LOAD, we performed a systematic literature search on PubMed, EMBASE and the Cochrane library using terms “*FERMT2*” or “fermitin family member 2,” “rs17125944,” “polymorphism,” and “Alzheimer's disease” or “AD.” The upper date was limited before February 2016. Since the restriction of language, only English articles were involved. And only the primary articles were included.

### Inclusion criteria

All the studies included should meet the following criteria: (1) assessing the relationship between rs17125944 and AD; (2) case-control studies; (3) NINCDS-ADRDA as the diagnostic criteria of AD; (4) support odds ratio (OR) with 95 % confidence interval (CI) or can calculated by sufficient data in studies.

### Data extraction

Following the inclusion criteria, data in available studies was extracted by two authors. We extracted the allelic odds ratio (OR) and 95% confidence intervals (CI), which were calculated by a logistic regression model. We also extracted other information, including author's name, publication year, ethnicity, numbers of cases and controls, proportion of female, mean age at onset in cases and mean age at examination in controls.

### Statistical analysis

The summary statistics from studies were combined by random effects (I-V heterogeneity) meta-analysis. Using the random (I-V heterogeneity) model, the pooled effect was estimated. The heterogeneity was calculated by with *I^2^* estimates (*I^2^* < 50% as “lack of heterogeneity”). All the statistical analyses were conducted by Stata 12.0 software.
